# Targeting Serglycin Prevents Metastasis in Murine Mammary Carcinoma

**DOI:** 10.1371/journal.pone.0156151

**Published:** 2016-05-25

**Authors:** Ananya Roy, Julia Femel, Elisabeth J. M. Huijbers, Dorothe Spillmann, Erik Larsson, Maria Ringvall, Anna-Karin Olsson, Magnus Åbrink

**Affiliations:** 1 Swedish University of Agricultural Sciences, Department of Biomedical Sciences and Veterinary Public Health, Box 7028, 75007, Uppsala, Sweden; 2 Uppsala University, Department of Medical Biochemistry and Microbiology, Box 582, 75123, Uppsala, Sweden; 3 VUMC—Cancer Center Amsterdam, Angiogenesis Laboratory, Dept. of Medical Oncology, De Boelelaan 1117, 1081 HV, Amsterdam, The Netherlands; 4 Uppsala University, Department of Immunology, Genetics and Pathology, Rudbeck laboratory, 751 85, Uppsala, Sweden; University of Patras, GREECE

## Abstract

In hematopoietic cells, serglycin proteoglycans mainly contribute to proper storage and secretion of inflammatory mediators via their negatively charged glycosaminoglycans. Serglycin proteoglycans are also expressed in cancer cells where increased expression has been linked to poor prognosis. However, the serglycin-dependent mediators promoting cancer progression remain to be determined. In the present study we report that genetic ablation of serglycin proteoglycan completely blocks lung metastasis in the MMTV-PyMT-driven mouse breast cancer model, while serglycin-deficiency did not affect primary tumour growth or number of mammary tumours. Although E-cadherin expression was higher in the serglycin-deficient primary tumour tissue, indicating reduced invasiveness, serglycin-deficient tumour cells were still detected in the circulation. These data suggest that serglycin proteoglycans play a role in extravasation as well as colonization and growth of metastatic cells. A microarray expression analysis and functional annotation of differentially expressed genes identified several biological pathways where serglycin may be important. Our results suggest that serglycin and serglycin-dependent mediators are potential drug targets to prevent metastatic disease/dissemination of cancer.

## Introduction

Serglycin proteoglycan is expressed by most hematopoietic cells, as well as endothelial cells and embryonic stem cells[1–5], where serglycin is involved in proper storage as well as secretion of inflammatory mediators, *e*.*g*. of proteases, histamine, cytokines and chemokines[6–10]. We have previously generated a serglycin-deficient mouse, viable and fertile, and showed that the deletion of serglycin indeed affects storage and activity of many leukocyte specific proteases. Although the deletion of serglycin affects many hematopoietic cell types the serglycin-deficient (SG^-/-^) mouse strain has a normal life span and seems immune-competent[6, 11, 12]. Serglycin is also expressed by cancer cells, *e*.*g*. in acute myeloid leukemia and myeloma as well as by nasopharyngeal carcinoma cells and breast cancer cells, where a correlation between the level of serglycin proteoglycan expression and a more aggressive tumour cell phenotype has been found[13–17]. Serglycin may protect the myeloma cells from complement attack via inhibition of both the classical and the lectin pathways[18]. On the cell surface serglycin can bind to CD44, and this complex promotes myeloma cell adhesion to collagen type 1 leading to up-regulation of matrix metalloproteinase (MMP) expression, which potentially could enhance the invasive and metastatic capacities[19–21]. The growth, invasion and metastasis of cancer cells depends on their interaction with various stromal cells and immune cells that infiltrate the tumour microenvironment[22]. To date studies investigating a potential role for serglycin in cancer have mainly examined tumour cells in culture or after xenogenic and heterotopic injection into immune-deficient mouse strains. We therefore addressed the importance of serglycin for tumour growth and metastasis in an orthotopic and spontaneous tumour mouse model by introducing serglycin-deficiency into the MMTV-PyMT mammary carcinoma model, in which lung metastases develop. To our knowledge this is the first study of serglycin in a spontaneous immune competent mammary tumour mouse model and we here report that expression of serglycin is essential for metastatic growth and dissemination.

## Materials and Methods

### Animal breeding, experimental groups and ethical statement

Congenic male FVB/n MMTV-PyMT+ transgenic mice[23] were crossed with congenic female SG^-/-^ C57BL/6 mice (N15)[6], and offspring was genotyped for the transgene. Two PyMT+ SG^+/-^ F1 males were selected and crossed with 2 females each of SG^-/-^ C57BL/6 (N15) to produce the littermates for the experimental breast cancer groups, that is PyMT+ SG^+/-^ and SG^-/-^ F2 female mice (now 25% FVB/n). The Neomycin resistance gene, *i*.*e*. the knockout cassette replacing exon 1, is still integrated in the genome of the serglycin-deficient mice, why we decided to breed for and analyse heterozygote SG^+/-^ mice, and not wild type SG^+/+^ mice, in comparison with the SG^-/-^ mice. All mice were scored blindly, *i*.*e*. the genotype was not known to the assessor until determined at endpoint, and the animals were euthanized when primary tumour burden reached 2 cm^3^ or when general health status was affected, *i*.*e*. body condition scoring[24] or when the tumour restrained the animal agility. The humane endpoint of individual tumour bearing F2 mice showed an age interval between 15–18 weeks, possibly due to the mixed genetic background. The Uppsala University Board of Animal Experimentation at Uppsala District Court approved all animal work (C 331/11) and all animals were handled in strict accordance with good animal practice as defined by the national and local animal welfare bodies. A maximum of 5 mice per cage were housed in IVCs (ca 501cm^2^ Macrolon IIL cages) with aspen shavings for bedding, paper for nesting and a small house of paper. A 12 hour light cycle was used and water and rodent chow were ad libitum.

### Antibodies

Primary antibodies: PyMT-antibody (ab15085, Abcam), anti-CD31 (ab24590, Abcam), anti-Ki67 (ab15580, Abcam), anti-cleaved caspase-3 (9661, Cell Signaling), anti-F4/80 Alexa Fluor 488 conjugate (MF48020, Life Technologies), anti-human fibrinogen (A0080, Dako), E-cadherin (sc-7870, Santacruz), CCL2/MCP-1 (sc-28879, Santacruz), anti-HGF (AF2207, R&D systems), anti-beta actin (BA3R, Thermo Scientific). Secondary antibodies: goat anti-mouse Alexa Fluor 488 (A11017, Invitrogen), goat anti-rabbit Alexa Fluor 594 (A11012, Invitrogen), goat anti-rat Alexa Fluor 680 (A21096, Invitrogen), goat anti-rat Alexa Fluor 568 (A11077, Invitrogen), donkey anti-rabbit Alexa Fluor 555 (A31572, Invitrogen), anti rabbit IR-Dye 800CW, anti goat IR-Dye 680CW, anti-mouse IRDye 800CW, anti-mouse IRDye 680CW

### Lectin perfusion, tissue preparation

Mice were anesthetized with avertin and then sacrificed with whole body perfusion using 10 ml PBS followed by fixation with 10 ml 2% paraformaldehyde. Some anesthetized mice also received FITC-conjugated tomato lectin (0.1 ml of 1 mg/ml in PBS) (FL-1171, Vector laboratories, Burlingame, CA) that was administered by retro orbital injection and left to circulate for 2 minutes before perfusion. Blood was collected during the initial 10 ml PBS perfusion and mixed with Trizol for RNA preparation. After perfusion tumours, lungs and liver were excised, collected in 30% sucrose solution and then stored frozen at -80°C until further use. For histological evaluation, tissues were dehydrated at 4°C in 30% sucrose in PBS, embedded in OCT compound and cryo-sectioned at 7–10 μm for collection onto Superfrost^®^ Plus microscope slides.

### Excision and morphological investigation of mammary tumours

Primary tumours were dissected and after removal of normal breast tissue, number of tumours and tumour weight were recorded to assess the total tumour burden. Morphological assessment and necrosis determination was done by an expert pathologist on 7 μm tumour sections stained with haematoxylin and eosin (H&E) in a blinded fashion. The tumours were in general distinctly circumscribed, but also extended outside colonizing normal mammary ducts. The tumour sections displayed variable amounts and different types of necrosis. Occasional large areas of dead tumour cells could be seen in few areas while in other parts the necrotic lesions were more of the comedo type that were localized in the central parts surrounded by vital cells. Between tumours a lucent stroma containing scanty inflammatory cells could be noticed. The grade of differentiation was usually moderate with a low-grade atypia among individual cells.

### Histology and Immunofluorescence

H&E staining was done to quantify metastasis in the lungs and necrosis in the primary tumours. Chloroacetate esterase staining was used to quantify mast cells in the primary tumour. Immunofluorescence staining was performed to visualize and quantify PyMT positive cells (1:500, antibody from Abcam) in lungs and primary tumours, as well as to assess proliferation (1:500, Ki67 antibody, Abcam), apoptosis (1:1000, cleaved caspase 3 antibody, Cell signaling), macrophage infiltration (1:200, F4/80 antibody, Life Technologies), endothelial cells (1:500, CD31 antibody, Abcam), and plasma leakage (1:500, fibrinogen antibody Dako) in the primary tumours. For immunostaining, sections were fixed with ice-cold methanol, washed and then blocked for 1 h at room temperature with 3% BSA in PBS followed by overnight incubation at 4°C with primary antibody diluted in blocking buffer. Hoechst (diluted 1:5000 in PBS) was used to visualize nuclei. Alexa Fluor secondary antibodies at 1:1000 dilutions were used for detection (45 min) of the primary antibodies. Prolonged washing between each step was done with PBS. Immunostained sections were mounted with Fluoromount G. Negative controls were done without primary antibody. High-resolution images for each section were taken using a Nikon 90i microscope and quantification of fluorescent regions in every section were done using ImageJ software. Approximately 1–3 tumours (depending on size) per mouse were selected for staining. For quantification of stained areas (% of 20X field using ImageJ64 software), 3–8 pictures of each tumour section depending on the tumour size were randomly taken with a 20X objective. Alternatively, cells staining positive for the respective marker were counted per field (20X) and the average of 7 to 10 fields calculated. For necrosis quantification from H&E stained sections, tumour areas and the percentage of necrotic and viable areas per section were assessed. Because the tumours were variable in size, percentage of necrosis was calculated in each tumour section.

### Western blots

Frozen tissue sections (100 mg) from the lungs and primary tumours were crushed in liquid nitrogen and suspended in RIPA buffer containing 2% Triton X-100 and proteinase inhibitors. The tissue lysate was left on ice for 15 min, centrifuged at 13,000 rpm for 20 min at 4°C, and supernatants were used for western blot analysis. Membranes were blocked with Odyssey^®^ blocking buffer (LI-COR Biosciences, part no: 927–40000) and all antibody dilutions were done in the blocking buffer. Washing of the membrane in between every step was done with PBS/0.01% Tween 20. IR-Dye secondary antibodies from LI-COR Biosciences were used for detection. Membranes were scanned using the Odyssey^®^ CLx imager (LI-COR Biosciences) scanner for visualization. Quantification of relative intensity was done using ImageJ software.

### Cytokine and angiogenesis array

Frozen tissue sections (50 mg) from the primary tumours were crushed in liquid nitrogen and suspended in PBS supplemented with protease inhibitors and stored in -20°C until used. Samples from 3 mice per genotype were pooled to perform the analysis of the cytokine (ARY006 from R&D systems) and angiogenesis (ARY015 from R&D systems) arrays. Analysis of the arrays was carried out according to the manufacturers instructions with a slight modification. In short, a secondary biotin labelled antibody was used to detect the primary antibody and streptavidin-Alexa was used for detection and visualization of the cytokine spots on the membranes. The filter layout and the area of the positive control spots was defined and used for all the other spots. All signal spots falling outside the filter layout were considered as artefacts. Quantification of the duplicate spots on the filters was done using ImageJ software as instructed by the manufacturers.

### RT-PCR detection of PyMT expression

RNA extraction from lung tissue, primary tumours, blood and liver followed the Trizol method. During the perfusion step blood was collected and mixed with Trizol. Frozen tissue sections (100 mg) were crushed in liquid nitrogen and suspended in Trizol. Ten ng of the purified total RNA was subjected to reverse transcriptase (Life Techonologies) with PyMT or GAPDH reverse primers and then PCR (KAPA biosystems) was performed using PyMT primers: forward 5’-CTGAGCCCGATGACAGCATA-3’, and reverse 5’-TCTTGGTCGCTTTCTGGATAC-3’. As baseline control of the RT-PCR mouse GAPDH primers were used: forward 5’-TGCCCCCATGTTTGTGATG-3’, and reverse 5’-TGTGGTCATGAGCCCTTCC-3’.

### Structural Analysis of HS and CS by Reverse Phase-Ion Pairing (RPIP)-HPLC

HS- and CS-chains were isolated from tumours and surrounding tissues and characterized essentially as described by Ledin et al., 2004[25]. In short, 40 mg dry weight of tissue samples were digested by consecutive proteolytic and nucleic acid degradation and GAGs purified by anion exchange chromatography on DEAE-gel. The CS-fraction of the purified GAG-pool was subjected to exhaustive degradation by chondroitinase ABC into CS-disaccharides and 10% of the sample analyzed, the remainder being cleaned from CS-disaccharides by another round of DEAE-chromatography before HS-chains were degraded into disaccharides by exhaustive cleavage with a mixture of heparin lyases I-III. CS- and HS-disaccharide pools, respectively, were analysed by reverse phase-ion pairing high performance chromatography (RPIP-HPLC) and quantified by post-column derivatization and detection of disaccharides as described[25]. Identity and amount of different disaccharide units was established by comparing the samples with HS- and CS-disaccharide standards run in parallel. As serglycin may be carrier of either glucosaminoglycan- (HS and heparin) or galactosaminoglycan- (CS and dermatan sulfate) chains we determined the content of these GAGs in tumour and normal tissue by exhaustive enzymatic degradation of the purified GAG-pools. Recovery of identified disaccharide units was quantified against known amounts of standard disaccharide units and related to dry tissue weight.

### Microarray expression and data analysis

RNA from tumour tissue was extracted using Trizol extraction protocol. RNA concentration was measured with ND-1000 spectrophotometer (NanoDrop Technologies, Wilmington, DE) and RNA quality evaluated with the Agilent 2100 Bioanalyzer system (Agilent Technologies Inc, Palo Alto, CA). 250 nanograms of total RNA from each sample were used to generate amplified and biotinylated sense-strand cDNA from the entire expressed genome according to the Ambion WT Expression Kit (P/N 4425209 Rev C 09/2009) and Affymetrix GeneChip® WT Terminal Labeling and Hybridization User Manual (P/N 702808 Rev. 6, Affymetrix Inc., Santa Clara, CA). GeneChip® ST Arrays (GeneChip® Mouse Gene 2.0 ST Array containing 33794 gene transcripts) were hybridized for 16 hours in a 45°C incubator, rotated at 60 rpm. According to the GeneChip® Expression Wash, Stain and Scan Manual (PN 702731 Rev 3, Affymetrix Inc., Santa Clara, CA) the arrays were then washed and stained using the Fluidics Station 450 and finally scanned using the GeneChip® Scanner 3000 7G. The raw data was normalized in the free software Expression Console provided by Affymetrix (http://www.affymetrix.com) using the robust multi-array average (RMA) method first suggested by Li and Wong in 2001[26]. Subsequent analysis of the gene expression data was carried out in the freely available statistical computing language R (http://www.r-project.org) using packages available from the Bioconductor project (www.bioconductor.org). In order to search for the differentially expressed genes between SG^+/-^ and SG^-/-^ groups an empirical Bayes moderated t-test was then applied, using the ‘limma’ package. To address the problem with multiple testing, the p-values were adjusted using the method of Benjamini and Hochberg[27]. Principal component analysis (PCA) and gene sets enrichment analysis (GSEA) were performed on the bioinformatic resource DAVID (the **D**atabase for **A**nnotation, **V**isualization and **I**ntegrated **D**iscovery)[28, 29]. The heat map and clustering was done using the Genesis software[30].

### Statistical analysis

Statistical analyses were performed using GraphPad Prism. Differences between SG^+/-^ and SG^-/-^ mice were assessed using the non-parametric two-tailed Mann Whitney test and differences indicated with their p values in the figure panels. Data are presented as mean ±SEM, except for S3 and S4 Figs where results are presented as mean pixel density.

## Results

### Serglycin is essential for metastasis to the lung

**Fig 1:**
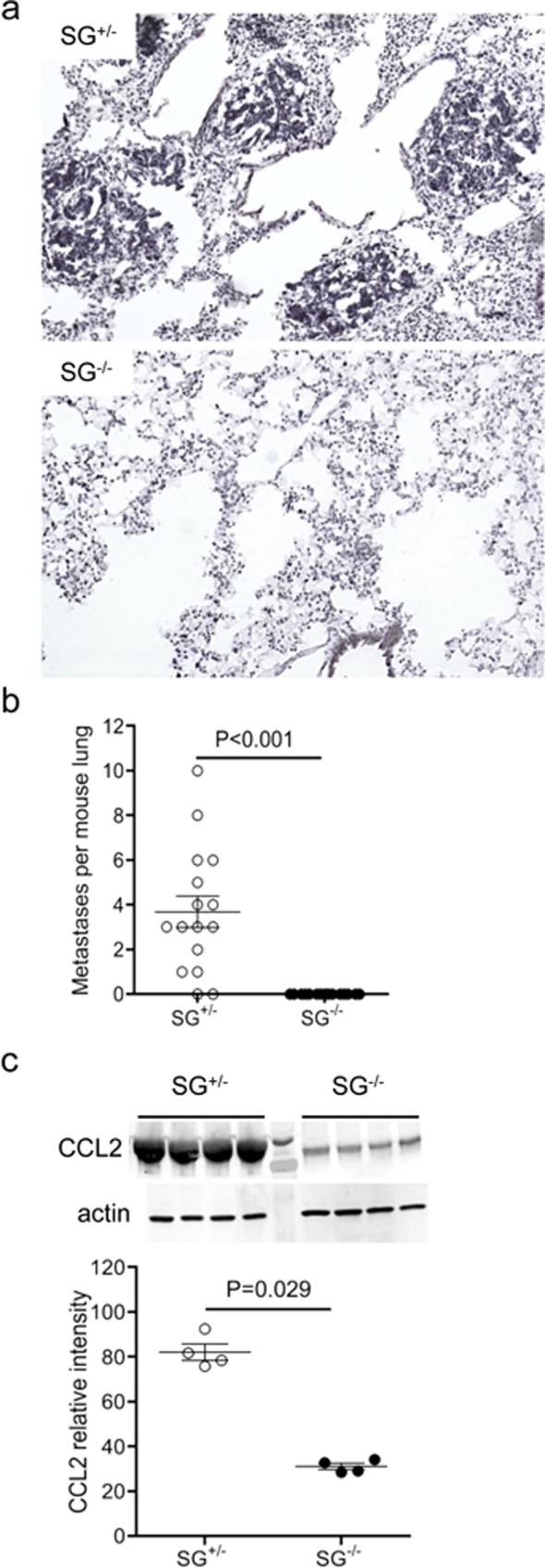
Serglycin is essential for metastasis to the lung. (**a**) representative photographs showing metastatic growth in the lung tissue of PyMT+ SG^+/-^ mice (upper photo) and the absence of metastases in PyMT+ SG^-/-^ mice (lower photo). (**b**) number of metastasis in the lungs quantified from H&E stained 7μm tumour sections (SG^+/-^ n = 16, and SG^-/-^ n = 16). Note that the SG^-/-^ mice displayed no lung metastases. To allow calculation of a statistical difference between SG^+/-^ and SG^-/-^ lung metastases, the SG^-/-^ mice value was set to 0.1. (**c**) upper panel: a representative western blot analysis of CCL2 levels in the lung with actin (middle panel) as a loading control. Lower panel: the relative intensity calculated from the representative western blot (SG^+/-^ n = 4, and SG^-/-^ n = 4). A total of 10 mice per genotype were analyzed by western blot and the CCL2 level was lower in all SG^-/-^ lungs. Using non-parametric two-tailed Mann Whitney test, the p values for the statistical differences between SG^+/-^ and SG^-/-^ are indicated in the figure.

To investigate the role of serglycin proteoglycans in metastatic breast cancer we crossed PyMT+ and SG^-/-^ mice. Lung is the major site for metastasis in the MMTV-PyMT model[23, 31, 32] and since serglycin has been implicated in aggressive metastatic cancer[15, 16] we first analysed the number of lung metastases in littermate PyMT+ SG^+/-^ and SG^-/-^ mice, respectively. Strikingly, not a single macroscopic or microscopic metastatic nodule was detected in cryo-sectioned lungs of SG^-/-^ mice when metastasis was quantified with H&E staining (Fig 1A and 1B) or with anti-PyMT immunohistochemistry (S1A and S1B Fig) whereas the SG^+/-^ mice had developed numerous metastatic nodules at endpoint. Furthermore, in line with the observed absence of metastasis in the SG^-/-^ lung tissue, CCL2-expression as an indication of metastasis-induced inflammation[33, 34] was significantly increased only in SG^+/-^ lung tissue (Fig 1C), suggesting that serglycin proteoglycan is essential for metastasis to the lung.

### Serglycin expression is not essential for primary tumour growth

**Fig 2:**
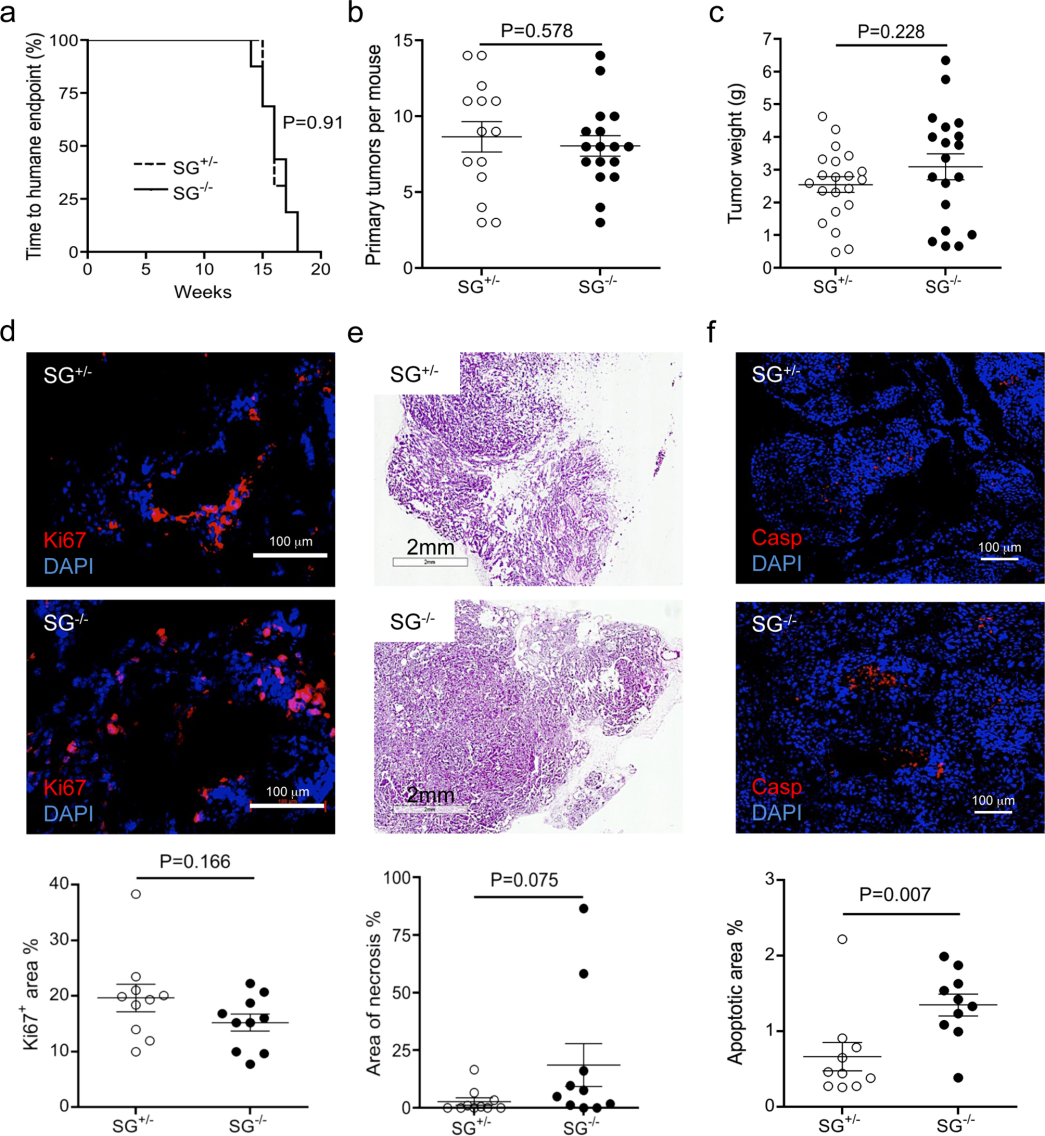
Tumour growth and tumour associated cell death in serglycin-deficient mice. (**a**) Kaplan-Meier survival curves of SG^+/-^ (n = 16) and SG^-/-^ (n = 16) mice. (**b**) Number of primary tumours per mouse, SG^+/-^ (n = 16) and SG^-/-^ (n = 16). (**c**) Total tumour weight per mouse, SG^+/-^ (n = 22) and SG^-/-^ (n = 19). (**d**) Representative photographs of immunhistochemical staining with Ki67 antibody of primary breast tumour tissue. Panel below: quantification of the Ki67 positive area (SG^+/-^ n = 10, and SG^-/-^ n = 10). (**e**) Representative photographs of primary breast tumour tissue with necrotic lesions. Panel below: quantification of the area of the necrotic lesions (SG^+/-^ n = 10, and SG^-/-^ n = 10). (**f**) Representative photographs of immunhistochemical staining with Caspase 3 antibody. Note that the signal is located in the center of the tumour foci. Panel below: quantification of the apoptotic area (SG^+/-^ n = 10, and SG^-/-^ n = 10). Using non-parametric two-tailed Mann Whitney test, the p values for the statistical differences between SG^+/-^ and SG^-/-^ are indicated in the figure.

To elucidate why serglycin-deficient mice appeared to be protected from lung metastases we next analysed the primary mammary tumours. The primary tumour growth observed in PyMT+ SG^+/-^ and SG^-/-^ mice was similar, with palpable tumours from 11–12 weeks of age. With a normal variation in the individual onset of tumour growth, the humane end point (defined by tumour volume, occasionally impaired movement, and general health status, *i*.*e*. body condition scoring) was reached between 14 and 18 weeks in both PyMT+ SG^+/-^ and SG^-/-^ mice. The variations in survival curve and age at the humane end point were similar in SG^+/-^ and SG^-/-^ mice (Fig 2A). Furthermore, the number of primary mammary tumours (Fig 2B) and total tumour weight (Fig 2C) were not significantly different between SG^+/-^ and SG^-/-^ mice. Histological analysis of tumour tissue from SG^+/-^ and SG^-/-^ mice showed a similar proliferation rate (Fig 2D), whereas a tendency to increased necrosis (Fig 2E) and a small but significantly increased apoptosis (Fig 2F) was found in the SG^-/-^ primary tumours.

To analyse vascular function, the mice were perfused with a fluorescently labelled (FITC) lectin that binds to the lumen of perfused blood vessels. Furthermore, vascular leakage was assessed by immunostaining for extravasated fibrinogen in the tissue. This was combined with immunofluorescent staining for CD31, a pan vascular marker. No significant difference in the extent of vascularization (CD31) (S2A and S2B Fig), perfusion (ratio FITC-lectin/CD31) (S2D Fig) or extravasated fibrinogen (ratio fibrinogen/CD31) (S2A–S2C Fig) was found between SG^+/-^ and SG^-/-^ primary tumours. In addition, when assessing the level of a panel of angiogenic factors on a multiplex antibody array some factors were reduced in the SG^-/-^ tumour tissue, but overall the serglycin-deficient tumour tissue showed no striking differences to the SG^+/-^ tumour tissue (S3 Fig). Taken together, our results suggest that serglycin is dispensable for primary tumour growth and functional vascularization of the primary tumour tissue in the PyMT mammary tumour model.

### Serglycin expression affects tumour inflammation levels

**Fig 3:**
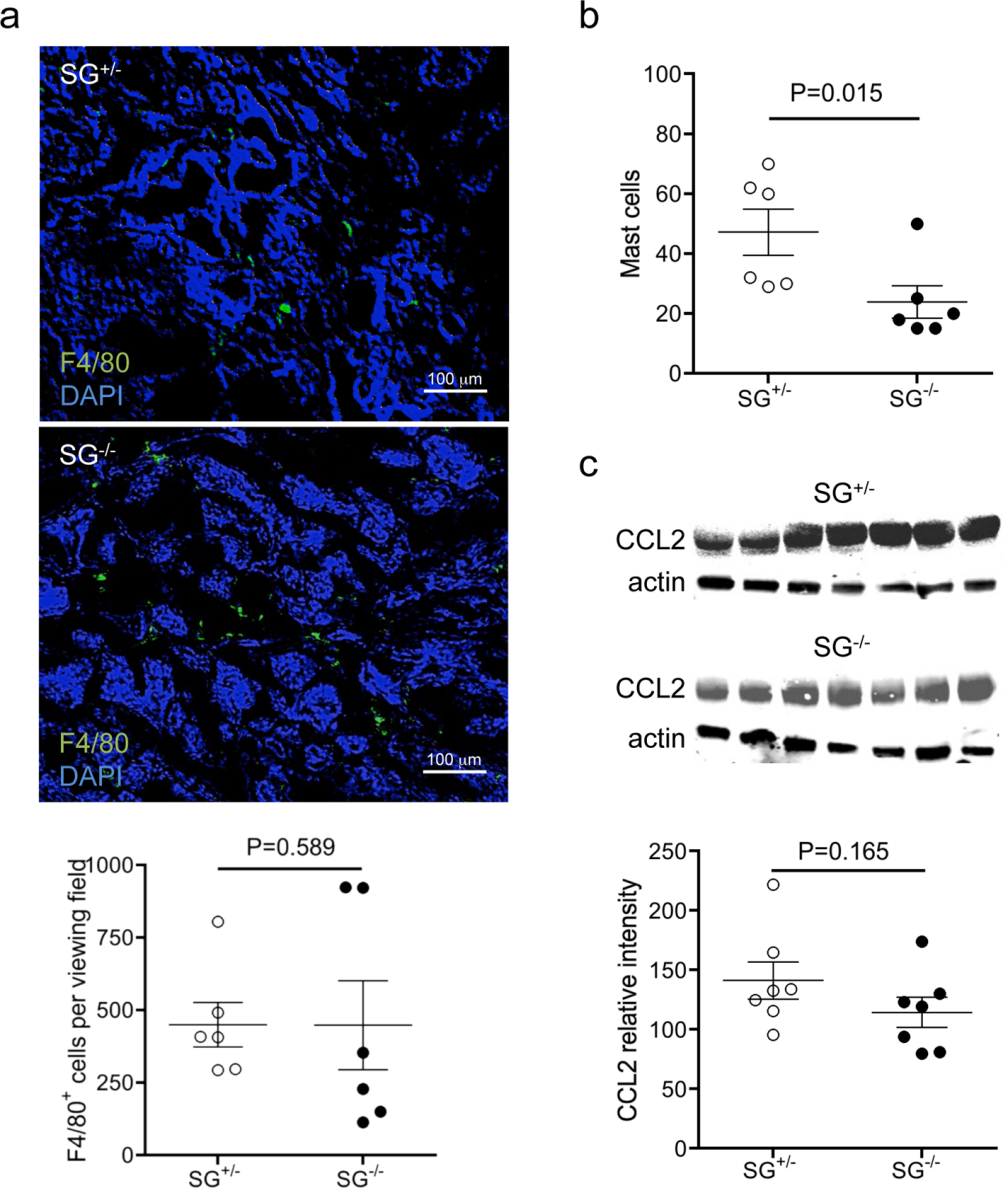
Inflammation levels in serglycin-deficient primary tumour tissue. (**a**) representative photographs (20X) of F4/80 positive macrophages (green) in breast tumour tissue (nucleus stained with DAPI, blue). Macrophages were quantified as mean ±SEM intensity of the positive staining (graph below) (SG^+/-^ n = 6, and SG^-/-^ n = 6). (**b**) Quantification of esterase positive mast cells in the primary breast tumour tissues (SG^+/-^ n = 6, and SG^-/-^ n = 6). (**c**) Western blot of CCL2 with actin as a loading control and the quantification of CCL2 levels normalised against actin levels, in the primary breast tumour tissues (SG^+/-^ n = 7, and SG^-/-^ n = 7). p values for the statistical differences between SG^+/-^ and SG^-/-^ are indicated in the graphs.

Inflammation is a potent driver of tumour progression and serglycin is strongly associated with inflammatory immune responses[35, 36]. Therefore we assessed if the deletion of serglycin altered the level of inflammation in the primary tumours. While the numbers of macrophages were similar in SG^+/-^ and SG^-/-^ primary tumours (Fig 3A), the number of mast cells in SG^-/-^ primary tumours was significantly decreased (Fig 3B). However, the pro-inflammatory cytokine CCL2 levels were not significantly different in the SG^-/-^ primary tumours compared to SG^+/-^ primary tumours (Fig 3C). Furthermore, the cytokine multiplex antibody array indicated that the levels of some inflammatory and immune regulatory cytokines were increased in the SG^-/-^ tumours (S4 Fig). The increased inflammatory cytokine levels found in the SG^-/-^ tumour tissue may be due to a decreased serglycin-dependent proteolytical degradation of these cytokines, possibly owing to reduced mast cell protease activity[4, 6]. Together, this indicates that serglycin affects the inflammatory response and may regulate the overall inflammation level in the mammary tumours.

### Deletion of serglycin does not change the primary tumour total glycosaminoglycan levels

Serglycin is one of the major proteoglycans expressed in leukocytes and altered proteoglycan expression[37] and changed levels of the glycosaminoglycan (GAG) chains chondroitin sulfate (CS) and heparan sulfate (HS) may potentiate growth factor signalling or the remodelling of extracellular matrix in cancer progression[38]. We therefore next speculated that the deletion of serglycin possibly could affect the total expression level of GAGs in mammary tumour tissue. The chromatography analyses indicated an increase of all detected CS and HS disaccharide units in tumour tissue compared to normal mammary tissue (S5A and S5B Fig), whereas the deletion of serglycin not significantly changed the overall CS and HS disaccharide content in the primary tumours (S5C and S5D Fig). This may be due to the fact that the extracted GAG-pools contain the total PG pool of GAGs, *i*.*e*. not only the GAGs from serglycin, and suggests that other proteoglycans potentially compensate for the lack of serglycin or that the contribution of serglycin proteoglycans to the total level of GAGs in tumour tissue is relatively small.

### Increased E-cadherin expression in serglycin-deficient tumour tissue

**Fig 4:**
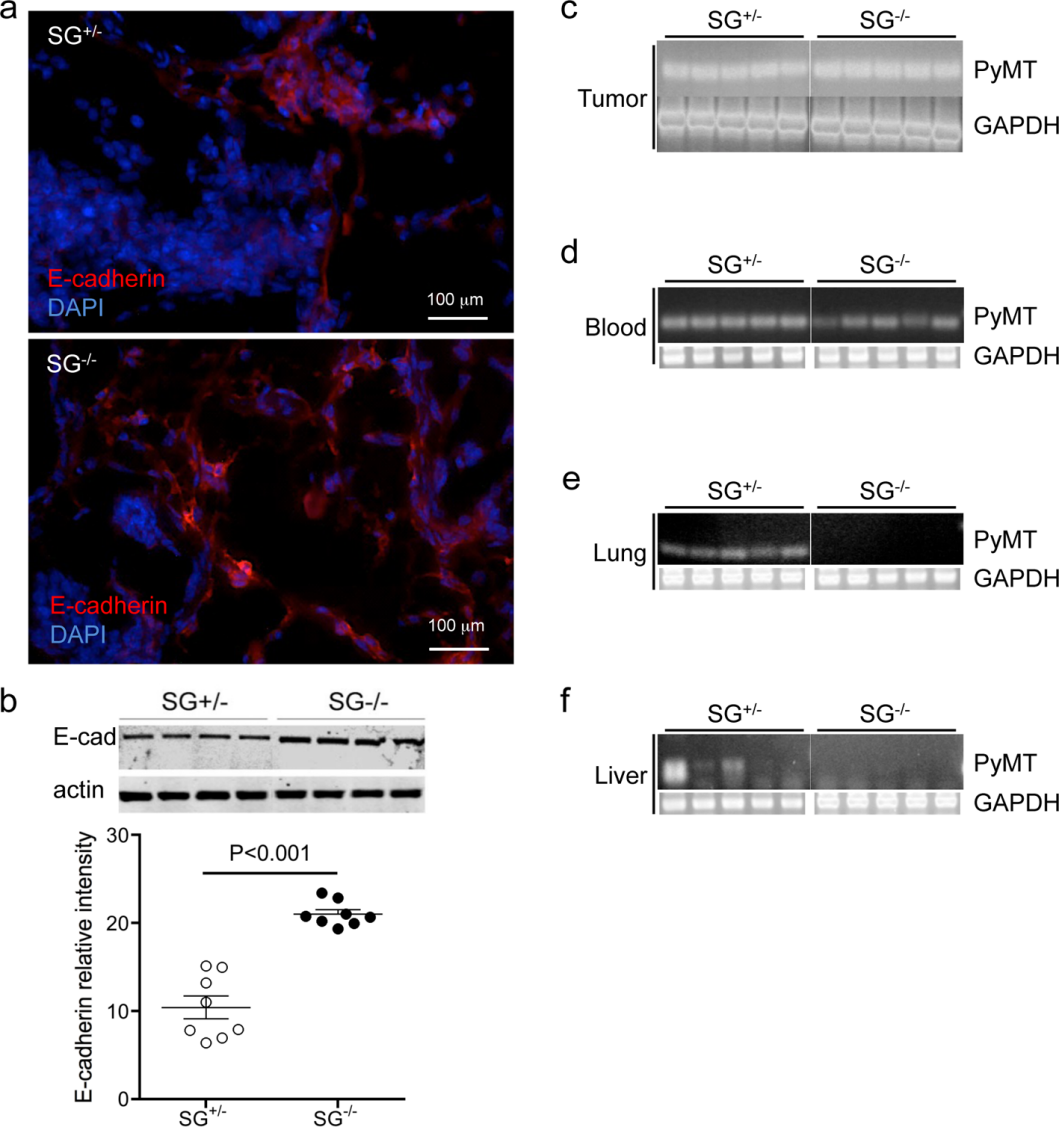
Epithelial-mesenchymal transition (EMT) in serglycin-deficient primary tumour tissue. (**a**) representative photographs of immunohistochemistry stained E-cadherin positive (red) SG^+/-^ and SG^-/-^ tumour tissue. (**b**) upper panel: a representative western blot analysis of E-cadherin levels in the tumour tissue. Actin was included as a loading control (SG^+/-^ n = 4, and SG^-/-^ n = 4). lower panel: levels of E-cadherin in tumour tissue quantified as the relative intensity from E-cadherin western blots (n = 8 per genotype). (**c-f**) show RT-PCR results of ten ng RNA extracted from different tissues. The PyMT-RNA levels in 5 individuals of each genotype were measured in (**c**) primary breast tumour tissue, (**d**) blood cells, (**e**) lung tissue and (**f**) liver tissue (SG^+/-^ n = 5, and SG^-/-^ n = 5). Note that no expression of PyMT was found in SG^-/-^ lung and liver. Amplification of GAPDH was used as a control of the RNA quality in each tissue. Using non-parametric two-tailed Mann Whitney test, the p values for the statistical differences between SG^+/-^ and SG^-/-^ are indicated in the figure.

Epithelial-to-mesenchymal transition (EMT) is a hallmark of invasive and metastatic tumour cells[39, 40] and involves the down-regulation of epithelial gene expression such as E-cadherin. To investigate the role of serglycin in EMT, we analysed the levels of E-cadherin in SG^+/-^ and SG^-/-^ primary tumours by Western blot and with immunohistochemistry. E-cadherin expression remained at a significantly higher level in the SG^-/-^ primary tumour tissue as compared with SG^+/-^ primary tumour tissue (Fig 4A and 4B), indicating a less invasive phenotype of serglycin-deficient tumour cells. To analyse the invasive capacity of the SG^-/-^ tumour cells we next investigated if circulating tumour cells were present in peripheral blood. The PyMT expression was assessed with RT-PCR and PyMT+ SG^+/-^ and SG^-/-^ primary tumour tissue served as a positive control (Fig 4C). Interestingly, PyMT+ cells were found in the blood of both PyMT+ SG^+/-^ and SG^-/-^ mice (Fig 4D), suggesting that tumour intravasation can occur in the absence of serglycin. In striking contrast, no RT-PCR signal was detected in the PyMT+ SG^-/-^ lung (Fig 4E) or the PyMT+ SG^-/-^ liver (Fig 4F), suggesting that even micro-metastases to the lung and liver were absent in mice lacking serglycin.

### Serglycin expression and the tumour transcriptome

**Fig 5:**
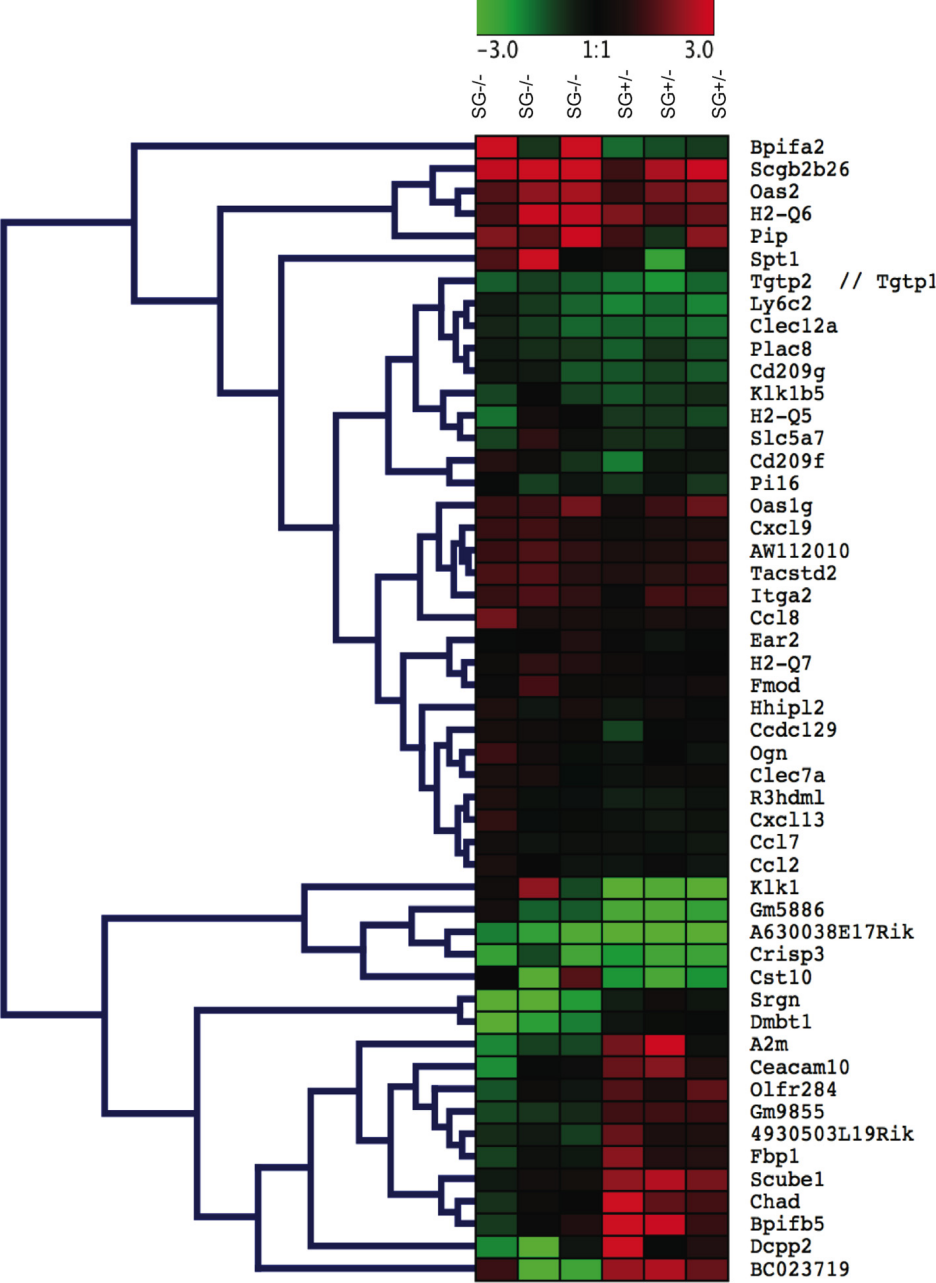
Heatmap of the top 51 differentially expressed genes in SG^+/-^ and SG^-/-^ tumour tissue (at log2 ≥ ±1, with p value < 0.05). The heatmap was constructed using Pearson Correlation and the tree structure built with Hierarchical Clustering. Red indicates maximum positive expression (+3) and green maximum negative expression (-3).

To identify potential serglycin-regulated mediators of tumour growth and metastasis, we performed a global gene expression analysis (GSE67806) of breast tumour tissues from three SG^+/-^ and three SG^-/-^ PyMT+ mice. A principal component analysis (PCA) of the data showed SG^+/-^ and SG^-/-^ RNA expression to be differentially clustered. The gene expression analysis identified 664 genes with a significantly altered expression (p-value <0,05) but when setting the expression level at a two-fold difference only 61 genes were differentially expressed in the SG^-/-^ tumour tissue compared to the SG^+/-^ tumour tissue. Of these 61 genes 51 were annotated genes (including serglycin). Importantly, the expression level of serglycin in the SG^-/-^ tumour tissue was 4.89 ±0.1 (log2), *i*.*e*. at background levels, whereas serglycin expression in the SG^+/-^ tumour tissue was 10.52 ±0.4 (log2). Of the 51 differentially expressed genes 13 were down-regulated and 38 were up-regulated in the SG^-/-^ mammary tumour tissue (Fig 5). Interestingly, of the two-fold down-regulated genes *Chad*, *Scube1* and *Ceacam10* are suggested to be involved in cellular adhesion. Of the two-fold up-regulated genes several are secreted, *e*.*g*. *Cst10* a proteinase inhibitor, *Crisp3* a matrix building protein, *Klk1* and *Klk1b5* which are trypsin-like proteases, and several chemokines *Ccl2*, *Ccl7*, *Ccl8*, *Cxcl9*, *and Cxcl13*. Since the protein level of E-cadherin was increased in the SG^-/-^ tumour tissue (Fig 4) we next checked the transcriptional level of *Cdh*1 on the microarrays. No significant difference of *Cdh1* expression between SG^+/-^ and SG^-/-^ tumour tissues was found (changed expression level, log2 = -0,128; P = 0,513). In contrast, although at a lower level other cadherins and protocadherins like *Cdh*3 (-0,65; P = 0,031) and *Pcdh*18 (-0,51; P = 0,021) were significantly down-regulated while *Cdh*5 (0,48; P = 0,012), *Pcdh*6 (0,60; P = 0,003), *Pcdh*12 (0,48; P = 0,031), *Pcdh*13 (0,55; P = 0,006), and *Pcdh*15 (0,58; P = 0,008) were significantly up-regulated. We also checked the transcriptional levels of cadherin-associated catenins but found no significant differences in expression between the SG^+/-^ and SG^-/-^ tumour tissues. However, the shift in the expression levels of all of these mediators requires further investigation at the proteome level to define the relationship to serglycin and their involvement in the metastatic process.

Finally, a gene set enrichment analysis (GSEA) of the 51 genes identified several cancer related processes with a significant enrichment score (ES): *e*.*g*. “Signal” and “Glycoprotein” (ES = 9.75); “Cell communication” and “Secreted” (ES = 5); “Chemotaxis” and “Inflammatory response” (ES = 3.74) (Table 1). These enrichment scores suggest that the deletion of serglycin affects the ability of the tumour cells to adhere to and signal to endothelial cells, and thereby interferes with the extravasation.

**Table 1:** Enrichment of gene sets in the SG^-/-^ tumour tissue, using DAVID.

Term	Benjamini	Enrichment Score	Genes	
			Up-regulated	Down-regulated
**Signal**	*1*.*4E-10*	*9*,*75*	*R3hdml*, *Ccl2*, *Ccl7*, *Ccl8*, *Cxcl13*, *Cxcl9*, *Crisp3*, *Ear2*, *Fmod*, *Hhipl2*, *H2-Q6*, *H2-Q7*, *Itga2*, *Klk1*, *Klk1b5*, *Ly6c2*, *Ogn*, *Pi16*, *Pip*, *Spt1*,*Tacstd2*	*Ceacam10*, *4930503L19Rik*, *Srgn*, *Scube1*, *A2m*, *Chad*
**Disulfide bond**	*1*.*1E-8*	*9*,*75*	*Clec12a*, *Clec7a*, *Ccl2*, *Ccl7*, *Ccl8*, *Cxcl13*, *Cxcl9*, *Crisp3*, *Ear2*, *Fmod*, *H2-Q6*, *H2-Q7*, *Itga2*, *Klk1*, *Klk1b5*, *Ly6c2*, *Ogn*, *Pip*, *Tacstd2*	*Scube1*, *Srgn*, *Dmbt1*, *A2m*
**Glycoprotein**	*2*.*6E-8*	*9*,*75*	*Clec12a*, *Clec7a*, *R3hdml*, *Ccl2*, *Ccl7*, *Cxcl9*, *Crisp3*, *Ear2*, *Fmod*, *Hhipl2*, *H2-Q6*, *H2-Q7*, *Itga2*, *Klk1*, *Klk1b5*, *Ly6c2*, *Ogn*, *Pi16*, *Spt1*, *Slc5a7*, *Tacstd2*	*A2m*, *Ceacam10*, *Srgn*, *Scube1*, *Chad*, *Dmbt1*
**Cell communication**	*1*.*9E-2*	*5*	*Ccl2*, *Ccl7*, *Ccl8*, *Cxcl9*, *Fmod*	*A2m*, *Ceacam10*, *Scube1*, *Chad*
**Secreted**	*2*.*8E-8*	*5*	*R3hdml*, *Ccl2*, *Ccl7*, *Ccl8*, *Cxcl13*, *Cxcl9*, *Fmod*, *Hhipl2*, *Ogn*, *Pip*, *Spt1*	*4930503L19Rik*, *A2m*, *Srgn*, *Chad*, *Dmbt1*, *Scube1*
**Signal transduction**	*3*.*8E-1*	*5*	*Ccl2*, *Ccl7*, *Ccl8*, *Cxcl9*, *Fmod*, *Olfr284*, *Ogn*, *Tacstd2*	*A2m*, *Ceacam10*, *Chad*, *Scube1*
**Inflammatory Response**	*2*.*6E-3*	*3*,*74*	*Clec7a*, *Ccl2*, *Ccl7*, *Ccl8*, *Cxcl13*, *Cxcl9*	*---*
**Chemotaxis**	*1*.*2E-4*	*3*,*74*	*Ccl2*, *Ccl7*, *Ccl8*, *Cxcl13*, *Ear2*	*---*
**Protease Inhibitor**	*1*.*4E-2*	*2*,*32*	*R3hdml*, *Cst10*, *Pi16*	*A2m*
**SCP-like Extracellular**	*1*.*3E-2*	*3*,*38*	*R3hdml*, *Crisp3*, *Pi16*	*---*
**Response to Stimulus**	*1*.*2E-2*	*3*,*83*	*Oas1g*, *Oas2*, *Clec7a*, *Tgtp2*, *Tgtp1*, *Ccl2*, *Ccl7*, *Ccl8*, *Cxcl13*, *Cxcl9*, *Ear2*, *H2-Q7*, *H2-Q6*, *H2-Q5*	*Gm9855*
**Carbohydrate Binding**	*1*.*2E-2*	*2*,*48*	*Clec12a*, *Clec7a*, *Cd209f*, *Cd209g*	*---*

## Discussion

A high level of expression of serglycin in tumour cells has been correlated to poor prognosis and an increased metastatic potential[15, 16]. The possible role of serglycin in metastatic dissemination was therefore studied in the transgenic MMTV-PyMT mouse model that develops orthotopic and spontaneous mammary tumours. This model is regarded to closely resemble the step-wise progression, morphology and molecular signature of human breast cancer[41]. The FVB/n MMTV-PyMT strain was crossed with the C57BL/6 serglycin-deficient (SG^-/-^) strain[6] and strikingly, the F2 PyMT+ SG^-/-^ mice seemed to be completely protected from pulmonary metastasis.

Although serglycin-deficient tumour cells were found in the circulation they failed to establish metastatic growth in lungs and liver at the experimental endpoint (Figs 1 and 4). In addition, only low levels of CCL2 were found in the lungs of PyMT+ SG^-/-^ mice suggesting that metastasis-induced inflammation were not present in the SG^-/-^ lungs. Since we could not detect any tumour cells in the SG^-/-^ lung by immunohistochemistry or by RT-PCR our results suggests that extravasation indeed may be blocked in the serglycin-deficient mice. However, at this time it could not be excluded that simply the metastatic latency was increased in the serglycin-deficient F2 mice (75% C57BL/6 and 25% FVB/n) as tumour onset is delayed at PyMT expression in congenic C57BL/6 mice compared to congenic FVB/n mice[42]. This latency depends on the genetic background of the mice and has been linked to regions on chromosomes 4, 7, and 9[43–45]. Importantly, however, both congenic C57BL/6 and FVB/n PyMT+ mice as well as F1 mice on a mixed FVB/n x C57BL/6 genetic background develop significant lung metastases[42, 46]. For ethical reasons, it was not possible to extend the period of observation because the sizes of the primary tumours reached the humane endpoint before eventual metastases occurred in the present F2 PyMT+ SG^-/-^ model. Nevertheless, to counteract eventual influence of a mixed genetic background or the gene-targeting cassette, which remains inserted in the serglycin-deficient mice, we strictly used heterozygote SG^+/-^ and homozygote SG^-/-^ PyMT+ female littermates to study how serglycin affects the metastatic potential in the model.

Targeting of serglycin with siRNA in human myeloma cells decreased cell adhesion *in vitro*, tumour cell growth *in vivo* and vascularization when injected subcutaneously into immunocompromised CB17 SCID mice, suggesting an essential role for serglycin in tumour growth and vascularization[17]. In contrast, serglycin-deficient mice showed increased tumour growth in the spontaneous and orthotopic insulinoma RIP-Tag2 cancer mouse model, possibly due to an improved tumour vessel functionality[47]. In the present PyMT+ mammary tumour mouse model SG^+/-^ and SG^-/-^ PyMT+ mice had similar numbers and weight of primary mammary tumours, expressed comparable levels of CD31 and displayed equal fibrinogen leakage, suggesting that genetic ablation of serglycin not affected the overall primary tumour growth or vessel formation. However, increased apoptosis and a trend towards increased necrosis were noted in the SG^-/-^ mammary tumours, which may explain why the overall tumour size was not significantly increased in the SG^-/-^ PyMT+ mice. In agreement with the present results, no correlation was found between serglycin expression and tumour numbers and size, when analysed in human hepatocellular carcinoma[48] or in nasopharyngeal carcinoma[15].

Previous studies in the serglycin proteoglycan deficient mouse strain has shown that the deletion impacts on the secretion of many tumour growth and metastasis promoting agents *e*.*g*. matrix metalloproteinases (MMPs), tumour necrosis factor (TNF)-alpha, platelet derived growth factor (PDGF), tissue type plasminogen activator, as well as several cytokines and chemokines[5, 10, 49, 50]. In the present model investigation of the primary tumours indicated that cancer related inflammation and epithelial to mesenchymal transition (EMT) were affected in mice lacking serglycin. While macrophage numbers and CCL2 levels did not differ between SG^+/-^ and SG^-/-^ mice, SG^-/-^ mice had decreased mast cell numbers and increased E-cadherin levels in the primary tumour tissue. However the increased E-cadherin levels was not matched with increased transcription of *Cdh*1 in the SG^-/-^ tumour cells suggesting that serglycin may have a role in E-cadherin degradation during tumour progression. In addition, we found small but significantly changed mRNA expression levels of other cadherins indicating that serglycin may be involved in the regulation of protocadherin and cadherin levels in the growing tumour tissue.

Furthermore, the higher E-cadherin expression in SG^-/-^ tumour cells and the complete absence of metastases in the PyMT+ SG^-/-^ lung tissue could reflect a lower degree of tumour cell intravasation into the circulation. This is however less likely because circulating tumour cells were detected in blood also in SG^-/-^ mice suggesting that intravasation was not dependent on serglycin. The failure of SG^-/-^ tumour cells to metastasize could instead be due to a reduced adhesion to matrix proteins like collagen and fibronectin, or a reduced protease activity affecting vascular permeability. Furthermore, serglycin is expressed by platelets[50] and it has been shown that platelets can bind to circulating tumour cells and thereby protect them from complement attacks[51]. Subcutaneous injection of SG^-/-^ and SG^+/-^ primary tumour cells in SG -competent and -deficient mice, respectively, will address if serglycin expression is required in the tumour cells or in the normal cell compartment to support metastasis. Our results point either to that the serglycin-deficient lung/liver microenvironment is unsuitable to support colonization and growth of the circulating SG^-/-^ tumour cells or that SG^-/-^ tumour cells cannot extravasate, and that other proteoglycans are unable to compensate for the lack of serglycin in these processes.

Deletion of serglycin affects strongly mast cell granulogenesis and granular retention/storage of the mast cell proteases[6, 52], and TNF-alpha secretion increases in LPS stimulated SG^-/-^ macrophages[10]. To identify serglycin-dependent mediators potentially involved in tumour progression, we next analysed the tumour tissue from SG^+/-^ and SG^-/-^ mice with multiplex antibody arrays. The PDGF levels, which may modulate tumour angiogenesis[53], were slightly decreased in the SG^-/-^ tumour tissue, while MMP-3, -8, -9 and TNF-alpha protein levels did not differ between SG^+/-^ and SG^-/-^ tumours. However, despite changed protein levels of some angiogenic and inflammatory mediators, the tumour progression, *i*.*e*. overall vascularization and tumour growth, was intact in the SG^-/-^ mice suggesting that expression of serglycin is not essential for primary tumour growth. The different effects of serglycin-deficiency found in the PyMT model and the RipTag2 model may thus reflect differences in the tumour growth conditions between the two models. Potentially, other proteoglycans may compensate for the serglycin-deficiency in growth and vascularization of the primary tumours[54, 55].

Global expression profiling has been extensively used to identify potential genes regulating tumour growth and metastatic dissemination. The global transcription analysis of primary tumour tissue allowed us to evaluate if the targeted deletion affects the expression of genes surrounding the serglycin gene at chromosome 10, ± 500kb. Careful examination (GSE67806) showed that the expression levels of genes upstream (Atoh7, Pbld1, Pbld2, Nnrnph3, Dna2, Slc25a16, Tet1, Snord98, Ccar1, Stox1, Ddx50, Ddx21) or downstream (Vps26a, Supv3I1, Hk1, Tacr2, Tspan15, Neurog3, Col13a1) of serglycin did not differ significantly between SG^+/-^ and SG^-/-^ tumours, indicating that heterozygotes are appropriate as controls. Interestingly, a metastatic gene signature involving around 17 genes has been described to arise already in primary spontaneous tumour cells[56–58]. While none of these metastatic signature genes were differently expressed in SG^-/-^ and SG^+/-^ primary tumours (GSE67806), the global transcription analysis identified 664 differentially expressed genes (p value <0.05) of which 61 were expressed at a two-fold difference. The top 51 genes, including serglycin, were used for the downstream gene sets enrichment analysis (GSEA) on the DAVID platform (Table 1). The GSEA showed that the deletion of serglycin affects several pathways that may play a role in tumour progression and metastasis of which four (“Signal”, “Cell communication”, “Secreted”, and “Inflammatory response”) have been identified in previous work on serglycin[6–10]. However, the exact role of serglycin in these pathways during cancer progression remains to be elucidated. Furthermore, the analysis by microarrays allowed for an initial screening of other suggested and potentially serglycin-dependent mediators at the transcriptional level. However, no significant difference in transcriptional levels of proteoglycan core proteins *e*.*g*. syndecans and glypicans, or glycosaminoglycan biosynthetic enzymes *e*.*g*. the synthases *Chsy*-1 and -3 as well as the different sulfotransferases (*Chst*1 up to *Chst*15) and the C-5 epimerases *Dse* and *Dsel*, or most MMPs were found when we selectively compared their expression level in SG^+/-^ and SG^-/-^ mammary tumours. However, although at lower transcriptional levels, MMP3 and 19 were significantly upregulated whereas MMP13 were significantly downregulated in the SG^-/-^ primary tumour tissue. The increased expression of MMP3 was found to cause a slight increase at the protein level in the SG^-/-^ tumour tissue (S3 Fig). The Affymetrix microarray analysis also showed that the CCL2 mRNA expression level was increased in the SG^*-/-*^ primary tumours (Fig 5 and Table 1), but no difference was found at the protein level (Fig 3 and S3 and S4 Figs) suggesting a serglycin-dependent effect on the CCL2 levels. In contrast, CCL5 and CXCL9 showed significantly higher mRNA expression levels and had increased protein levels in the SG^-/-^ tumour tissue (Fig 5 and S4 Fig). Thus, the gene expression differences found in this study calls for a detailed proteomic analysis of the SG^-/-^ and SG^+/-^ tumour cells.

In summary this is the first report on the role of serglycin in tumour progression using an immune competent spontaneous and orthotopic mammary tumour mouse model that develops lung metastases. Our results show that serglycin is essential for the metastatic capacity of mammary tumour cells. Although we found tumour cells in the circulation, no tumour cells were detected in lungs of the serglycin-deficient PyMT+ mice, suggesting that extravasation is blocked. Hence, the role of serglycin in colonisation and metastatic growth cannot be assessed in the current model. Our finding warrants further investigations on the role of serglycin in metastatic cancer growth, with the aim to characterize the serglycin-dependent metastatic pathways and to identify novel drug targets.

## Supporting Information

S1 Fig Anti-PyMT immunohistochemistry stained lungs of SG^+/-^ and SG^-/-^ mice. Representative photographs showing metastatic growth in the lung tissue of PyMT+ SG^+/-^ mice (upper photos) and the absence of metastases in PyMT+ SG^-/-^ mice (lower photos). Nucleus was stained with DAPI, blue. Note that the staining produces some background, *i*.*e*. in bronchiole.
(TIF)

S2 Fig Normal vascularization in serglycin-deficient primary breast cancer tumours. (**a**) To assess tumour vasculature numbers, tumour sections were immunofluorescently stained against CD31 (green, left photos) and fibrinogen (red, center photos) and merged with DAPI stain (blue, right photos). (**b**) The CD31 positive staining was quantified as percent of total area (DAPI, blue) (SG^+/-^ n = 10, and SG^-/-^ n = 10). (**c**) Quantification of plasma leakage presented as ratio of fibrinogen (red) positive staining/CD31 (green) positive staining (SG^+/-^ n = 7, and SG^-/-^ n = 7). (**d**) Quantification of FITC-lectin perfused vessels in the primary tumours (SG^+/-^ n = 5, and SG^-/-^ n = 4). p values for the statistical differences between SG^+/-^ and SG^-/-^ are indicated in the graphs.
(TIF)

S3 Fig Multiplex Angiogenesis Antibody Array detection of the relative levels of angiogenesis-related proteins in SG^+/-^ and SG^-/-^ tumour tissue. Samples from 3 mice per genotype (SG^+/-^ and SG^-/-^) were pooled for the array. Quantification of the relative intensities was done using ImageJ. (white columns show SG^+/-^ and black columns SG^-/-^)
(TIF)

S4 Fig Multiplex Cytokine Antibody Array detection of the relative levels of cytokines and chemokines in SG^+/-^ and SG^-/-^ tumour tissue. Samples from 3 mice per genotype (SG^+/-^ and SG^-/-^) were pooled for the array. Quantification of the relative intensities was done using ImageJ. (white columns show SG^+/-^ and black columns SG^-/-^)
(TIF)

S5 Fig Glycosoaminoglycan analysis of the primary tumour tissue in SG^+/-^ and SG^-/-^ mice. The total GAG and disaccharide content in primary tumour tissue (n = 3) compared against normal breast tissue (n = 2), showing total CS in (**a**) and HS in (**b**). In (**c**) and (**d**) the total GAG and disaccharide content was compared in SG^+/-^ (n = 3) and SG^-/-^ (n = 3) primary tumours with comparison of CS in (**c**) and HS in (**d**).
(TIF)
