# Supplementary figures and images for: Targeting Serglycin Prevents Metastasis in Murine Mammary Carcinoma
Source: PLoS One. 2016 May 25;11(5):e0156151. doi: 10.1371/journal.pone.0156151 (PMC4880347; doi:10.1371/journal.pone.0156151)

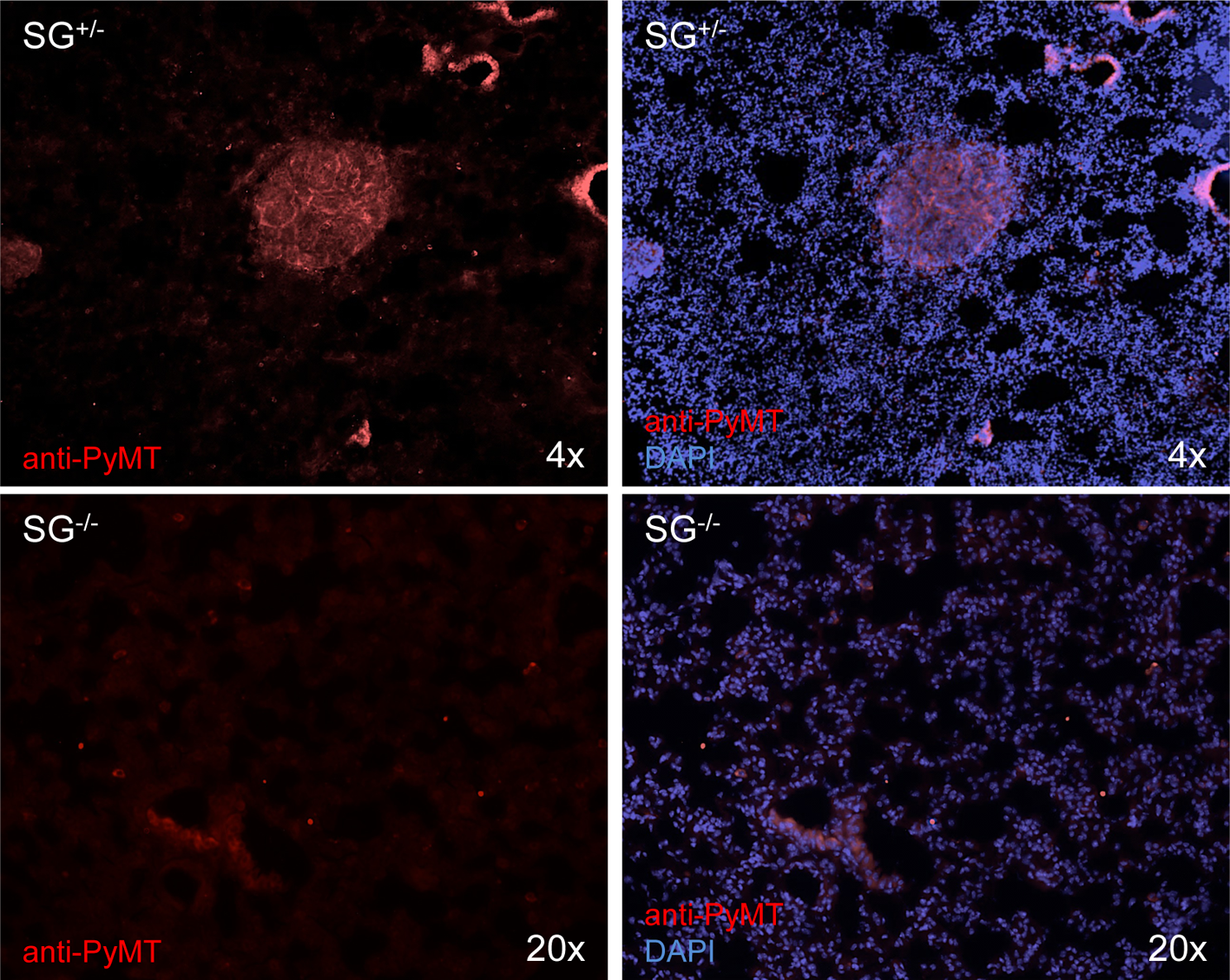

Supplement: S1 Fig — Representative photographs showing metastatic growth in the lung tissue of PyMT+ SG+/- mice (upper photos) and the absence of metastases in PyMT+ SG-/- mice (lower photos). Nucleus was stained with DAPI, blue. Note that the staining produces some background, i.e. in bronchiole. (TIF) [file pone.0156151.s001.tif]

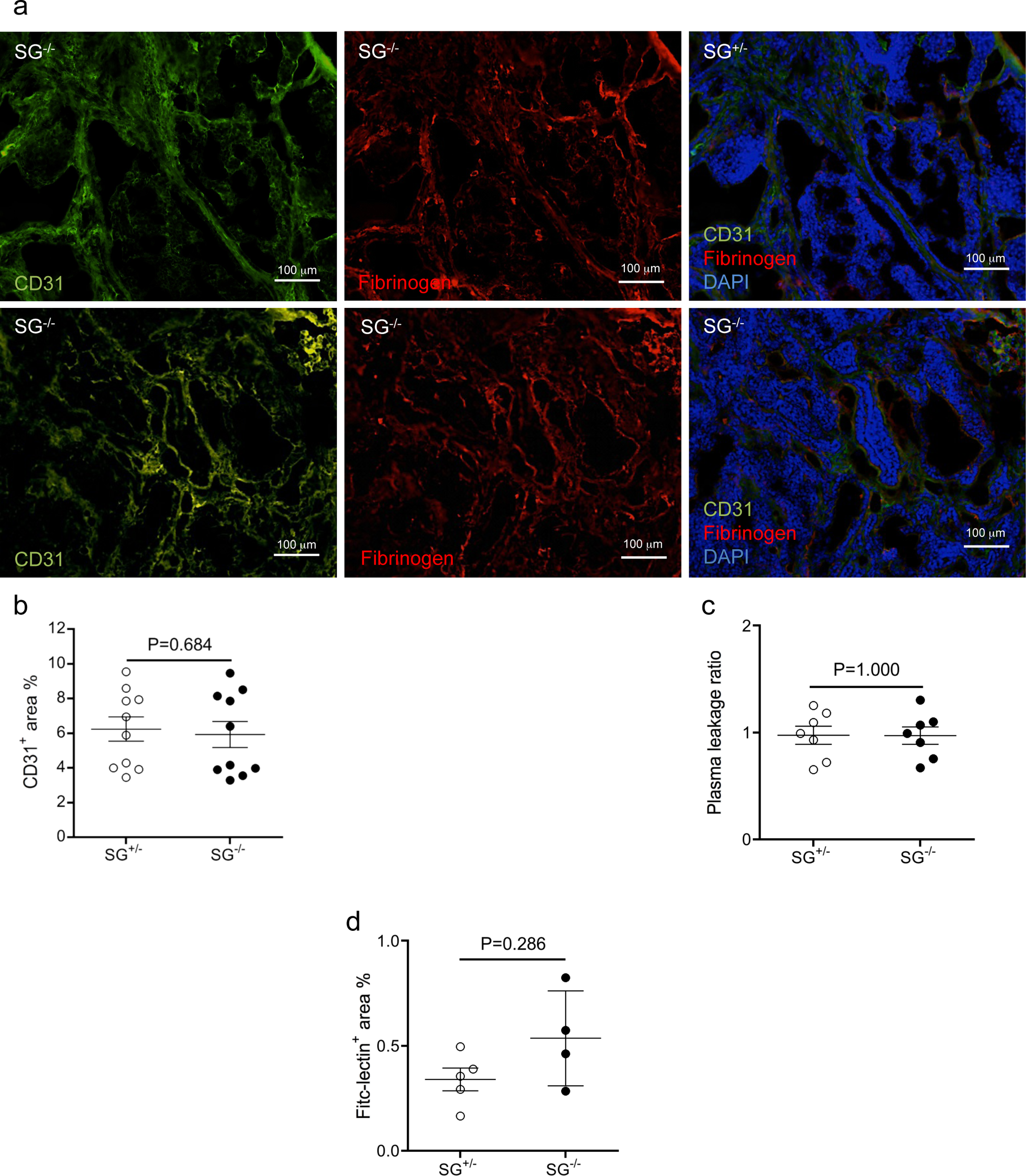

Supplement: S2 Fig — (a) To assess tumour vasculature numbers, tumour sections were immunofluorescently stained against CD31 (green, left photos) and fibrinogen (red, center photos) and merged with DAPI stain (blue, right photos). (b) The CD31 positive staining was quantified as percent of total area (DAPI, blue) (SG+/- n = 10, and SG-/- n = 10). (c) Quantification of plasma leakage presented as ratio of fibrinogen (red) positive staining/CD31 (green) positive staining (SG+/- n = 7, and SG-/- n = 7). (d) Quantification of FITC-lectin perfused vessels in the primary tumours (SG+/- n = 5, and SG-/- n = 4). p values for the statistical differences between SG+/- and SG-/- are indicated in the graphs. (TIF) [file pone.0156151.s002.tif]

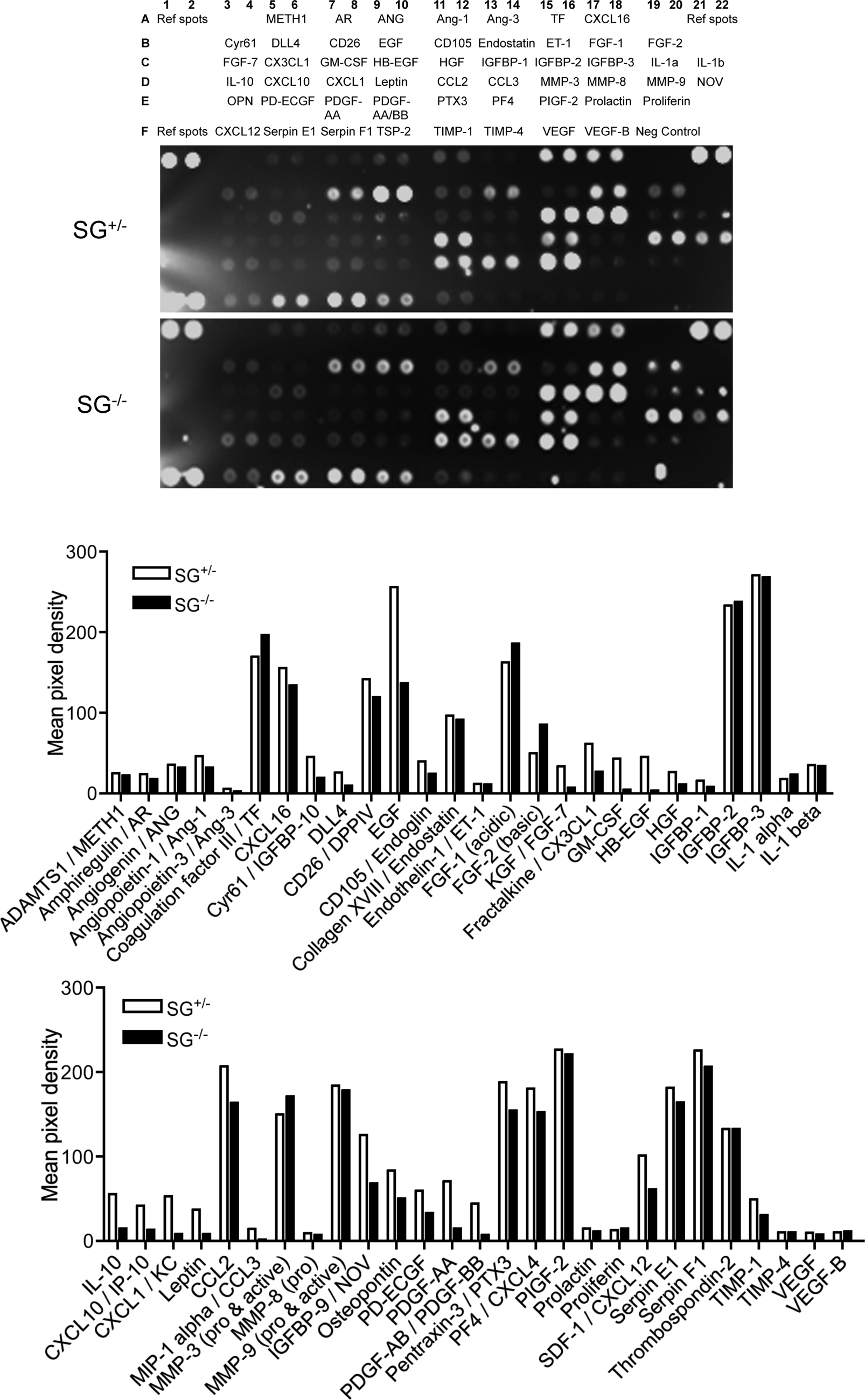

Supplement: S3 Fig — Samples from 3 mice per genotype (SG+/- and SG-/-) were pooled for the array. Quantification of the relative intensities was done using ImageJ. (white columns show SG+/- and black columns SG-/-) (TIF) [file pone.0156151.s003.tif]

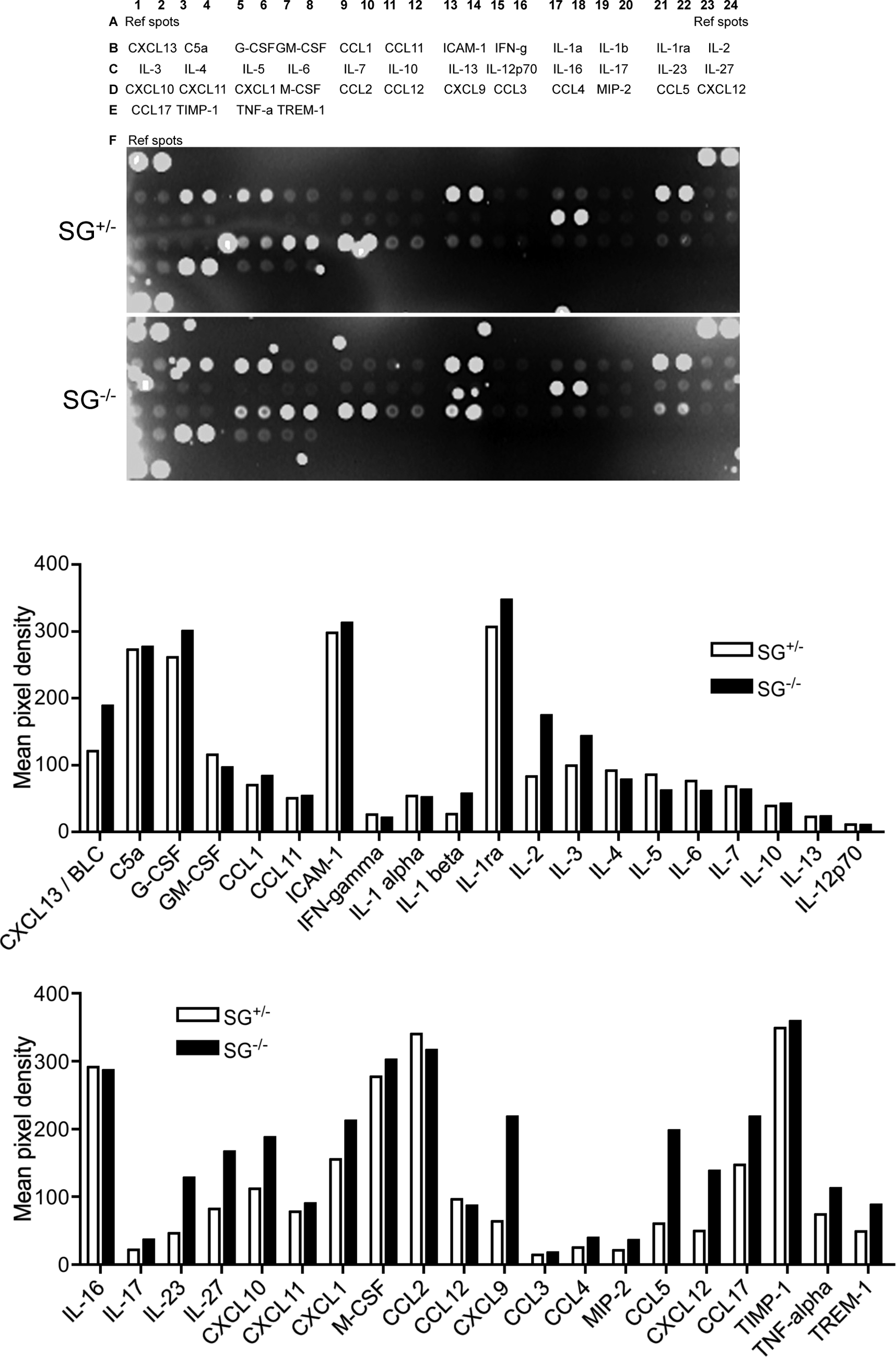

Supplement: S4 Fig — Samples from 3 mice per genotype (SG+/- and SG-/-) were pooled for the array. Quantification of the relative intensities was done using ImageJ. (white columns show SG+/- and black columns SG-/-) (TIF) [file pone.0156151.s004.tif]

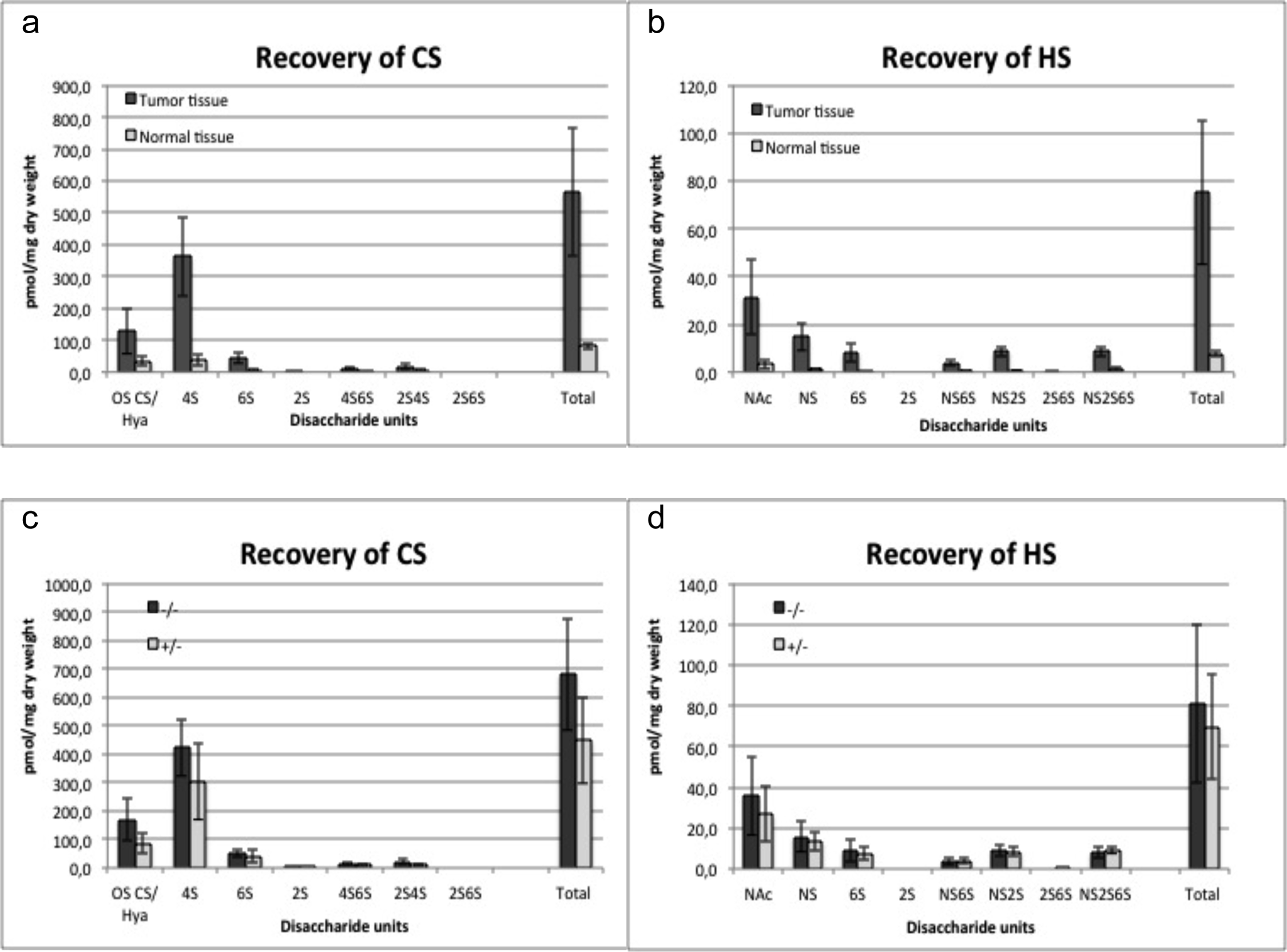

Supplement: S5 Fig — The total GAG and disaccharide content in primary tumour tissue (n = 3) compared against normal breast tissue (n = 2), showing total CS in (a) and HS in (b). In (c) and (d) the total GAG and disaccharide content was compared in SG+/- (n = 3) and SG-/- (n = 3) primary tumours with comparison of CS in (c) and HS in (d). (TIF) [file pone.0156151.s005.tif]
